# The Importance of Being Presented: Target Validation by Immunopeptidomics for Epitope-Specific Immunotherapies

**DOI:** 10.3389/fimmu.2022.883989

**Published:** 2022-04-06

**Authors:** Jonas P. Becker, Angelika B. Riemer

**Affiliations:** ^1^Immunotherapy and Immunoprevention, German Cancer Research Center (DKFZ), Heidelberg, Germany; ^2^Molecular Vaccine Design, German Center for Infection Research (DZIF), Partner Site Heidelberg, Heidelberg, Germany

**Keywords:** immunopeptidomics, T cell epitope, tumor antigen, HLA class I, neoantigen, cancer immunotherapy

## Abstract

Presentation of tumor-specific or tumor-associated peptides by HLA class I molecules to CD8^+^ T cells is the foundation of epitope-centric cancer immunotherapies. While often *in silico* HLA binding predictions or *in vitro* immunogenicity assays are utilized to select candidates, mass spectrometry-based immunopeptidomics is currently the only method providing a direct proof of actual cell surface presentation. Despite much progress in the last decade, identification of such HLA-presented peptides remains challenging. Here we review typical workflows and current developments in the field of immunopeptidomics, highlight the challenges which remain to be solved and emphasize the importance of direct target validation for clinical immunotherapy development.

## Introduction

Immunotherapy has been established as the fourth pillar of cancer treatment. The immune system’s ability to discriminate self from non-self constitutes the basis of all immunotherapeutic interventions. Essential for this discrimination process are small peptides, termed epitopes, presented *via* human leukocyte antigen (HLA) complexes at the cell surface allowing constant monitoring of both intra- and extracellular protein expression and thus the cell’s health state. HLA class I molecules are expressed by virtually all nucleated cells and present short peptides (8-15 amino acids) of mainly intracellular origin. HLA class I:peptide complexes are surveyed by CD8^+^ T cells and allow them to identify and, ultimately, eliminate infected or malignant cells. HLA class II expression is restricted to professional antigen presenting cells and mainly responsible for the display of longer peptides (up to 25 amino acids) of extracellular origin to CD4^+^ T cells (1). Collectively, all HLA-presented peptides are termed immunopeptidome.

**Figure 1:**
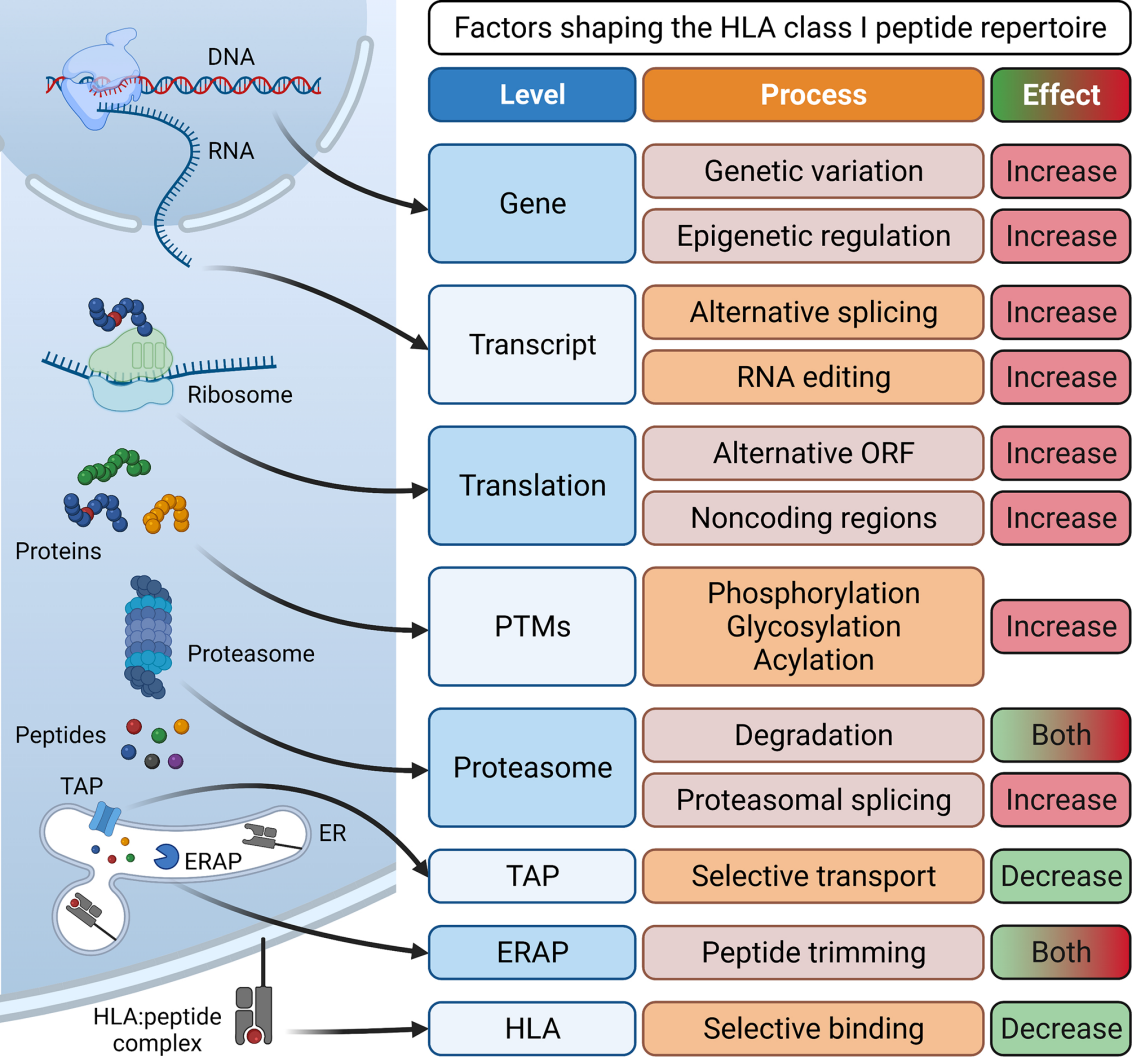
Processes involved in antigen processing and presentation and their effect on the generation of a diverse HLA class I peptide repertoire. Peptides for surface presentation by HLA class I molecules are generated by transcription of DNA into mRNA and subsequent translation into proteins. Here, processes such as genetic variation and epigenetic regulation at the gene level, alternative splicing and RNA editing at the transcript level, the translation of alternative open reading frames (ORFs) and non-coding regions and post-transcriptional modifications (PTMs) can enlarge the peptide repertoire. Eventually, proteins are degraded by the proteasome, generating peptides. Here, proteasomal splicing can increase peptide diversity while proteasomal degradation can also reduce the size of the repertoire by destroying some potential HLA binders. Finally, peptides are selectively conveyed *via* the transporter associated with antigen processing (TAP) into the lumen of the endoplasmic reticulum (ER). Here, some peptides are further trimmed by ER-associated aminopeptidases (ERAP) and then selectively associate with specific human leukocyte antigen (HLA) complexes. Eventually, stable HLA:peptide complexes are transported to the cell surface for interaction with CD8^+^ T cells. Created with BioRender.com.

Essential for the development of epitope-specific cancer immunotherapies such as therapeutic vaccines or T cell receptor (TCR)-transgenic T cells is the identification of tumor-associated antigens (TAAs) or tumor-specific antigens (TSAs). **Tumor-associated antigens** show low expression or are absent in healthy, adult tissue (2). TAAs are categorized into developmental antigens (normally expressed only during embryonal development), cancer-testis antigens (normally expressed only in reproductive tissue), overexpressed antigens, and post-translationally modified antigens. Examples of well-studied TAAs include the developmental antigen CEA (carcinoembryonic antigen), the cancer-testis antigens of the MAGE (melanoma-associated antigen) family and NY-ESO-1, the overexpressed TAAs epidermal growth factor receptor 2 (HER2) in breast cancer or CD19 in B cell malignancies, and the post-translationally modified antigen MUC-1 (3). Importantly, immune responses against TAAs are limited by central tolerance mechanisms and often lack complete specificity to cancer cells. In contrast, **tumor-specific antigens** represent optimal targets since they are exclusively expressed on cancer cells but not on healthy host cells and are therefore not subject to central tolerance. TSAs can arise from genomic protein-coding alterations such as single nucleotide variants, gene fusions, or InDel mutations (4–9). Collectively, these TSAs have been termed “neoantigens”. More recently, the antigenic repertoire has been extended by “non-canonical tumor antigens”, for which it is not yet clear if they are tumor-associated or truly tumor-specific, and therefore their possible clinical relevance is highly debated. They arise from alternative mRNA splicing (10, 11), RNA editing (12, 13), usage of alternative transcription start sites or reading frames (14, 15), as well as cryptic peptides that are produced by proteasomal splicing and lack exome evidence (16, 17). Other sources of tumor antigens include peptides with post-translational modifications (18, 19) and peptides of viral (20, 21) or bacterial origin (22) presented in a tumor-associated or tumor-specific manner (**Figure 1**).

The main strategies used today to identify neoantigens are based on next-generation sequencing data and subsequent prioritization of candidates using *in silico* HLA binding prediction. At best, peptide immunogenicity is further validated using *in vitro* assays, however, whether these peptides are actually presented *via* HLA class I molecules on the target cell surface is rarely confirmed. This is all the more important, because sequencing data and even assessment of a potential source protein’s expression level are not directly reflected in epitope presentation. In fact, biogenesis of HLA class I-presented peptides does not obey the “law of mass action”, meaning that the proportion of presented epitopes does not represent the amount of the source proteins within the cell’s proteome (23). While this allows the efficient sampling of a large part of a cell’s proteome and the timely recognition of malignant or infected cells, it hampers the identification of neoantigens solely based on next generation sequencing and *in silico* HLA binding prediction. Additionally, peptides derived from non-canonical open reading frames constitute a non-neglectable part of the immunopeptidome, which, however, is not completely assessable by basic next-generation sequencing approaches (24, 25).

Currently, the only available technique to provide a direct proof of presentation for clinically relevant target epitopes is mass spectrometry-based immunopeptidomics.

## Stages of a Typical Immunopeptidomics Workflow

### Sequencing Approaches to Define Tumor Antigens (Setting the “Search Space”)

In order to identify tumor-specific peptide sequences in immunopeptidomics assays, these sequences first have to be identified from next-generation sequencing data and included into the “search space” [recently reviewed in (26)]. At the DNA level, data generated by whole-genome sequencing (WGS) and whole-exome sequencing (WES) are commonly used for this task. While WES is less cost-intensive than WGS, it only allows the identification of tumor antigens derived from protein-coding parts of the genome. Analyses deploying RNA sequencing technologies further allow the identification of potential tumor antigens derived from other sources such as alternative splicing, intron retention, RNA editing and other non-canonical sequences. Additionally, RNA sequencing provides information on gene expression levels, which, however, should be handled with caution when prioritizing tumor antigen candidates (see above and “Lessons learned from immunopeptidomics”). Ribosome profiling (Ribo-seq) allows the identification of transcripts undergoing active translation. Using the inherent periodicity of the obtained reads it is possible to generate sample-specific *de novo* reference proteomes including previously unannotated ORFs potentially harboring tumor antigens (15, 27).

Importantly, all these workflows usually generate large datasets of potential tumor antigens, exceeding the capacity to test all of them in laborious and time-consuming cellular assays. Thus, computational revision of the “search space” is typically performed as a next step.

### HLA Ligand Processing and Binding Prediction Approaches (Revising the “Search Space”)

Since there are a large number of biological processes involved in antigen processing and presentation, there are just as many computational tools to predict if a given peptide is likely to be presented at the cell surface. For HLA class I ligands, ubiquitinated proteins are digested in the cytosol by the proteasome, which can exist in the canonical form and, induced by interferons, as immunoproteasome. Given the different cleavage specificities of these subtypes, some tumor antigens require one or the other form to be generated efficiently (28, 29). Several algorithms exist for the prediction of proteasomal cleavage sites, for example, NetChop20S (30) and ProteaSMM (31) which are trained on *in vitro* degradation data. After their release from the proteasome into the cytosol, peptides may be further trimmed by cytosolic peptidases such as e.g. TPPII. NetChopCterm (30) is trained on actual HLA ligand data taking into account these other proteases involved in antigen processing and therefore performs best for prediction of HLA ligand processing (32). Resulting peptides are transported into the endoplasmatic reticulum (ER) *via* transporter associated with antigen processing (TAP) molecules, which again show selectivity for certain peptide characteristics. As for proteasomal cleavage, several algorithms have been developed to model peptide transport into the ER (31, 33, 34). In the ER, peptides can be further trimmed by ER-resident peptidases such as endoplasmatic reticulum aminopeptidases (ERAP), before being loaded onto HLA class I molecules. Both, polygeny and polymorphism of the HLA class I gene locus ensure that a large peptide repertoire can be displayed to CD8^+^ T cells by forming different peptide binding groves. Conserved amino acids at the anchor positions of the bound peptides allow sequence clustering to identify HLA binding motifs (35–37), defining HLA supertypes and developing algorithms for HLA binding prediction. Since the binding affinity of a peptide to a given HLA molecule is thought to be the most critical determinant of actual peptide presentation, much effort has been invested in the development and constant improvement of such algorithms (38–40). While most of the initial algorithms were based solely on binding affinity data, newer iterations have been much improved by the incorporation of eluted ligand data generated by mass spectrometry (41, 42). Nevertheless, several studies reported discrepancies between predicted binders and actually presented peptides (43–45). Moreover, shortlisting of candidates by HLA binding predictions creates an *a priori* bias and might exclude actually presented tumor-specific epitopes (46, 47). Both considerations are particularly important when utilizing predictions for rare HLA alleles with little to no data available to train the respective algorithms. Ultimately, tumor-specific peptides presented by HLA class I molecules at the cell surface have to be recognized by CD8^+^ T cells in order to induce tumor cell elimination. As not all presented tumor-specific epitopes trigger the necessary T cell activation, recent modeling approaches focused on the prediction of immunogenicity based on peptide presentation and recognition characteristics (48, 49) or by structural modeling of HLA:peptide and HLA:peptide:TCR complexes (50, 51). A number of computational pipelines implementing next-generation sequencing data and prediction of HLA ligand processing and binding have been proposed and used to prioritize tumor antigen candidates [(52, 53); for an extensive list see (54)].

### MS Methodologies for Immunopeptidomics (Exploring the “Search Space”)

The field of immunopeptidomics first emerged in the early 1990s when peptides presented by HLA molecules were characterized using Edman degradation (35, 55) and mass spectrometry (56). More recently, the field gained new momentum with the introduction of high-sensitivity MS instrumentation and the finding that T cells, reactivated by immune checkpoint blockade, specifically recognize tumor-specific epitopes (57–59). In contrast to the enormous technical advancements, the basic principles of immunopeptidomics sample preparation have remained largely the same (60). HLA-presented peptides are either dissociated nonspecifically by mild acid elution (MAE) or HLA:peptide complexes are purified by immunoprecipitation (IP) using a specific antibody with subsequent separation of peptides and HLA molecules. Sample preparation by IP represents the currently preferred method due to its high specificity and increased yield compared to MAE (61).

Different MS acquisition methods have been applied for the interrogation of the immunopeptidome and the identification of tumor antigens: data-dependent acquisition (DDA), targeted data acquisition, and more recently data-independent acquisition (DIA). As most immunopeptidomic experiments aim at the identification of yet unknown epitopes, discovery-driven DDA is the most commonly used method for the generation of tandem mass spectra. The obtained data is then used for the identification of peptides and their amino acid sequence either by comparison to theoretical mass spectra of all precursors in a database with a similar mass-to-charge (m/z) ratio, by *de novo* sequencing, or a combination of the two approaches. Using DDA, several thousand unique HLA class I-presented peptides can be identified. Since the heuristic selection and subsequent fragmentation of peptide precursors depend on their abundance, low abundant peptides (such as most tumor-specific epitopes) are prone to be missed by DDA, and DDA lacks reproducibility and quantitative accuracy. In contrast, targeted acquisition methods such as selective/multiple reaction monitoring (S/MRM) and parallel reaction monitoring (PRM) are considered the gold standard for identification and quantification, in particular of low abundant peptides. In targeted approaches, only predefined peptides are selected for fragmentation. A predefined subset of fragments in the case of S/MRM or all fragments in the case of PRM are then detected and used for identification and quantification. The major drawbacks of these targeted approaches are the need for *a priori* knowledge of the analyte of interest, *i.e.* the epitope, as well as the labor-intensive pre-selection of suitable transitions in the case of S/MRM analyses. Targeted analyses are thus restricted to the detection of pre-defined peptide subsets of limited size and do not offer comprehensive investigation of the complete immunopeptidome. More recently, DIA has been introduced for the analysis of the immunopeptidome leading to improvements in both reproducibility and quantification compared to DDA (62–64). For DIA, all precursors within a defined mass-to-charge window are subjected to fragmentation in an unbiased manner generating highly convoluted mass spectra. This process is iterated across the full mass-to-charge range to generate a “digital map” of the analyzed sample. To identify peptides from such mass spectra, they are compared to a spectral library containing fragmentation and retention time information of the peptides of interest. The generation of a high-quality spectral library usually requires preceding extensive DDA analysis of the same or a related sample. More recently, the introduction of library-free approaches (65–67) and tools for *in silico* prediction of mass spectra (68–70) have enabled comparable performances without the need of an experimental spectral library.

## Considerations Before and After Mass Spectrometry

Besides the major improvements in instrumentation, several other aspects of a typical immunopeptidomics workflow have been subjected to optimization. For example, the analysis of the immunopeptidomes from HLA-monoallelic cell lines does not require deconvolution of HLA binding motifs and was shown to improve predictive power of HLA binding algorithms trained on the respective data (71). Other studies utilized secreted and C-terminally tagged HLA class I molecules to increase the purification yield and reduce the need for detergents during sample preparation (72, 73). In the future, similar approaches for rare and poorly investigated HLA alleles might uncover their binding motifs and further improve HLA binding predictions. Additionally, it has been reported that various parameters such as the usage of different detergents for cell lysis or choice of peptide purification methods can both affect the yield and repertoire of identified peptides (74). Studies employing heavy-labelled HLA:peptide complexes estimated peptide losses of up to 99% during immunoprecipitation (75, 76). Several novel approaches for the sample preparation process have been proposed in order to improve both sensitivity and reproducibility, including a high-throughput immunoprecipitation protocol (77), a semi-automated workflow utilizing TMT labeling (78), as well as a microfluidic-based HLA enrichment protocol (79).

For proteogenomics, and mass spectrometry-based immunopeptidomics in particular, extremely inflated search spaces such as those created for example from three- or six-frame translations of RNA-seq and Ribo-Seq data can significantly increase the rate of false-positive identifications (80). This rate can be controlled by target-decoy approaches. Here, decoy sequences (*e.g.* reversed canonical protein sequences) are included into the database and score distributions of target and decoy matches can be used to separate false from true identifications and calculate false discovery rates (81–83). The confidence in peptide identifications can be increased by combining the results of different search engines (15) or by multi-round database search approaches searching first against canonical protein databases and subsequently against larger databases containing potential neoepitopes (47). MS-Rescue (84) and DeepRescore (85) are machine learning-based algorithms which increase both sensitivity and reliability of peptide identifications using information such as peptide binding motifs, retention times and mass spectra predictions. Moreover, tools to create HLA-specific, and therefore smaller, peptide databases exist (86, 87) and can be used to decrease the probability of false-positive identifications. Additionally, the quality of an immunopeptidomics dataset can be assessed *a posteriori* by performing HLA binding predictions as well as peptide sequence clustering (36) or by correlating observed retention times with calculated hydrophobicity indices (88) or predicted retention times (89), respectively. Finally, the ultimate proof of a correct peptide identification is provided by the comparison of retention times and mass spectra recorded from synthetic peptides with those obtained from the biological sample (21, 90, 91).

## Lessons Learned From Immunopeptidomics

Much progress has been made in the field of immunopeptidomics in the last decade, however, some limitations still apply. There are tens of thousands of potential binders per HLA allele and although it has been shown that there are “hotspots” for the generation of HLA class I-presented peptides (92, 93), it is unlikely that each and every HLA class I-presented peptide will be identified (94, 95). In the case of mutation-derived neoepitopes, it has been postulated that on average only one such neoepitope is identifiable per 1.8x10^8^ non-synonymous mutations and per 1.1x10^4^ unique HLA class I-presented peptides (96), emphasizing the need for increased sensitivity both in sequence variant calling and mass spectrometry. The latter is best illustrated by comparing the already impressive sensitivity of modern mass spectrometers in the attomolar range to the sensitivity of CD8^+^ T cells, with early reports suggesting that a single to a few hundred HLA class I-presented peptides per cell are sufficient to trigger an immune response (97–99), effectively rendering T cells 10^3^ to 10^6^ times more sensitive than current generation MS instruments (100). Although often used to prioritize neoepitope candidates, RNA expression data should be handled with caution as several studies showed that there is no clear correlation between the transcriptome and the immunopeptidome (15, 101, 102). Moreover, peptides have been identified without the detection of the corresponding transcript, for example if they are derived from long-lived proteins, defective ribosomal products (DRiPs) or short-lived transcripts that are rapidly degraded by nonsense-mediated RNA decay (101, 103–105).

## Immunological Validation of Target Neoepitopes After Immunopeptidomics

**Figure 2:**
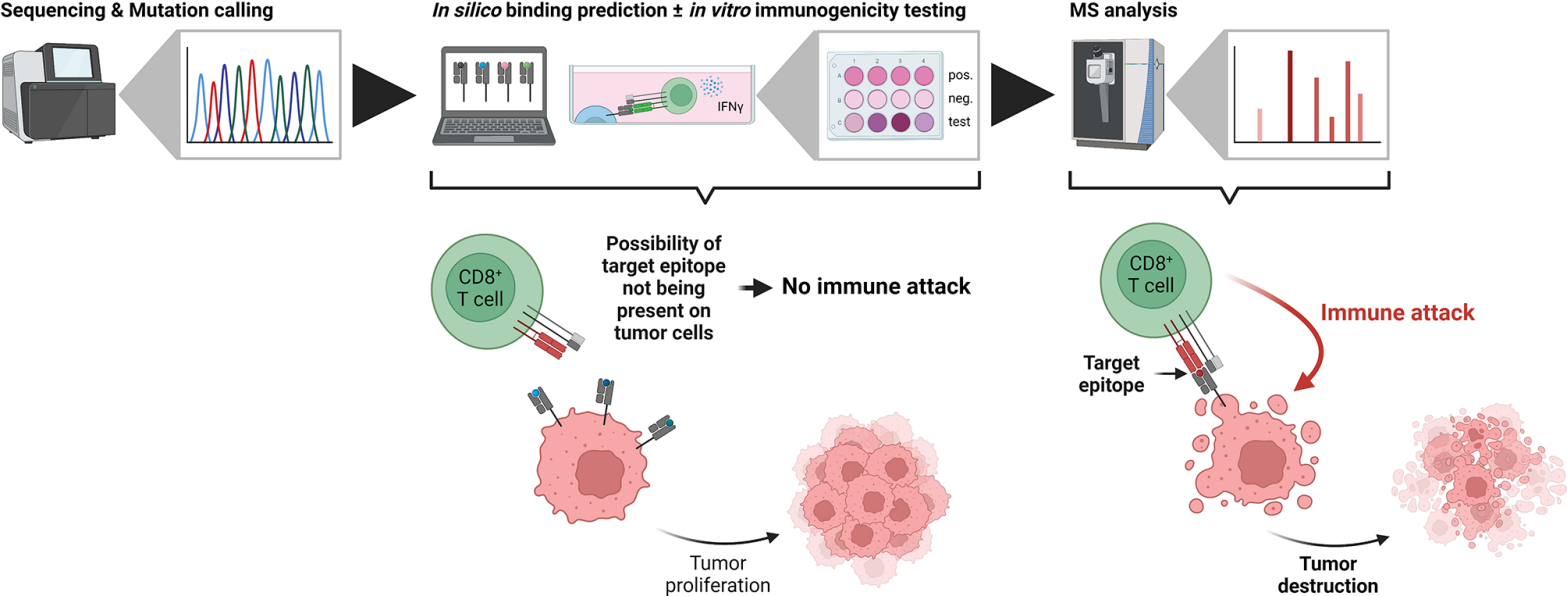
The importance of being presented. In typical neoantigen identification workflows, candidate tumor antigens are defined from next generation sequencing data (left side). Subsequent *in silico* binding predictions and *in vitro* immunogenicity tests identify potential tumor neoepitopes, which, however, are not necessarily presented on the tumor cells themselves. If the target epitope is not present, CD8^+^ T cells cannot identify the transformed cells and the tumor proliferates (middle). In contrast, mass spectrometry-based immunopeptidomics identifies truly presented and actionable epitopes. Immunotherapies targeting these epitopes lead to an immune attack by CD8^+^ T cells and tumor destruction (right side). Created with BioRender.com.

Using sequencing-based approaches for epitope prioritization, there usually remain hundreds to thousands of candidates depending on the mutational load of the investigated sample – an amount that is unfeasible for validation using classical approaches such as *in vitro* binding or T cell killing assays. In contrast, mass spectrometry-based identification of target epitopes greatly reduces the number of candidates and additionally limits them to those actually processed and presented by HLA class I molecules at the cell surface. For the clinical application of any epitope-based therapy, proof of immunogenicity, *i.e.* T cell reactivity, represents the ultimate prerequisite. It can be assessed either functionally by the detection of cytokine secretion using ELISA or ELISPOT assays (106, 107), by the direct detection of T cells recognizing a given epitope using peptide-MHC multimers (108, 109) or by *in vitro* expansion of T cells in combination with TCR sequencing (110, 111). Other considerations before the clinical application of any epitope-based therapy should include the clonal expression and tumor specificity, as well as the “similarity-to-self” of the target epitope. Clonal expression has recently been shown to be a critical determinant for tumor rejection in both mice and humans (112–114). True tumor specificity is of particular importance to avoid off target effects as exemplified by severe neurological toxicity in a clinical trial utilizing MAGE-A3 TCR-engineered T cells (115). Likewise, “similarity-to-self” should be considered for the selection of actionable target epitopes to both avoid off-target effects and to mount effective T cell responses (116). The recent introduction of the HLA Ligand Atlas (117) provides a comprehensive resource of HLA-presented peptides for various benign tissues which should be considered when selecting tumor-associated target epitopes including those arising from non-canonical sources. Despite the importance of proving the immunogenicity of a given candidate epitope, it has to be emphasized that it only can be of therapeutic benefit if it is actually processed and presented by cancer cells (**Figure 2**).

## Concluding Remarks

Mass spectrometry-based immunopeptidomics currently is the only unbiased methodology for the identification of target epitopes actually HLA-presented on the cell surface and should be utilized when selecting targets for clinical applications such as adoptive cell transfer and cancer vaccines. Although mass spectrometry is still comparatively insensitive relative to T cells and thus might miss some target epitopes, several studies involving immunopeptidomics have been performed or are under active investigation in clinical trials. Vaccination with IMA901, consisting of multiple tumor-associated epitopes identified and validated by the MS-centric XPRESIDENT approach (118), showed an association between T cell responses to tumor-associated epitopes and increased survival in patients with renal cell carcinoma (119). A personalized dendritic cell vaccine approach was used for the delivery of mutation-derived neoepitopes in melanoma patients and shown to promote both expansion as well as diversity of epitope-specific T cells. HLA class I-mediated presentation of the selected epitopes was confirmed by immunopeptidomics (120). Utilizing a warehouse vaccine concept with 14 HLA class I and class II tumor-associated epitopes, iVAC-XS15-CLL01 is currently under clinical investigation for the treatment of chronic lymphocytic leukemia (121). The GAPVAC-101 trial investigated the combination of a warehouse vaccine and a fully personalized vaccine in glioblastoma patients and showed the induction of both CD8+ and CD4+ T cell responses (122).

Ideally, future therapeutic approaches will target epitopes that are shared between patients. Here, tumor-associated epitopes as well as viral epitopes are intriguing targets, however also tumor-specific epitopes can be targeted in larger cohorts for some indications such as microsatellite-instable cancers (123). The increasing number of non-canonical sources for HLA class I-presented peptides as well as the identification of bacteria-derived peptides presented by HLA molecules in a tumor-specific manner potentially represent a rich pool of targetable epitopes (22, 124). Additionally, new therapeutic approaches targeting multiple HLA allotypes will increase the number of patients benefiting from such interventions (125).

Much progress has been made in the field of immunopeptidomics within the last decade and future technical and computational advancements overcoming the previously described remaining limitations and further pushing the limits of peptide detection will certainly strengthen the importance of mass spectrometry-based identification of target epitopes in the future. As the ultimate and most direct proof of epitope presentation remains reserved for immunopeptidomics, it is expected to become even more integral to the development of epitope-specific immunotherapies.

## Author Contributions

JB and AR wrote this manuscript together and both approved the submitted version.

## Conflict of Interest

The authors declare that the research was conducted in the absence of any commercial or financial relationships that could be construed as a potential conflict of interest.

## Publisher’s Note

All claims expressed in this article are solely those of the authors and do not necessarily represent those of their affiliated organizations, or those of the publisher, the editors and the reviewers. Any product that may be evaluated in this article, or claim that may be made by its manufacturer, is not guaranteed or endorsed by the publisher.
